# Does ethnicity influence dementia, stroke and mortality risk? Evidence from the UK Biobank

**DOI:** 10.3389/fpubh.2023.1111321

**Published:** 2023-04-14

**Authors:** Bruno Bonnechère, Jun Liu, Alexander Thompson, Najaf Amin, Cornelia van Duijn

**Affiliations:** ^1^Nuffield Department of Population Health, University of Oxford, Oxford, United Kingdom; ^2^REVAL Rehabilitation Research Center, Faculty of Rehabilitation Sciences, Hasselt University, Diepenbeek, Belgium; ^3^Technology-Supported and Data-Driven Rehabilitation, Data Science Institute, Hasselt University, Diepenbeek, Belgium

**Keywords:** risk score, ethnicity, dementia, stroke, epidemiology

## Abstract

**Introduction:**

The number of people with dementia and stroke is increasing worldwide. There is increasing evidence that there are clinically relevant genetic differences across ethnicities. This study aims to quantify risk factors of dementia, stroke, and mortality in Asian and black participants compared to whites.

**Methods:**

272,660 participants from the UK Biobank were included in the final analysis, among whom the vast majority are white (*n* = 266,671, 97.80%), followed by Asian (*n* = 3,790, 1.35%), and black (*n* = 2,358, 0.84%) participants. Cumulative incidence risk was calculated based on all incident cases occurring during the follow-up of the individuals without dementia and stroke at baseline. We compared the allele frequency of variants in Asian and black participants with the referent ethnicity, whites, by chi-square test. Hierarchical cluster analysis was used in the clustering analysis. Significance level corrected for the false discovery rate was considered.

**Results:**

After adjusting for risk factors, black participants have an increased risk of dementia and stroke compared to white participants, while Asians has similar odds to the white. The risk of mortality is not different in blacks and white participants but Asians have a decreased risk.

**Discussion:**

The study provides important insights into the potential differences in the risk of dementia and stroke among different ethnic groups. Specifically, the study found that black individuals had a higher incidence of dementia and stroke compared to white individuals living in the UK. These findings are particularly significant as they suggest that there may be underlying factors that contribute to these differences, including genetic, environmental, and social factors. By identifying these differences, the study helps to inform interventions and policies aimed at reducing the risk of dementia and stroke, particularly among high-risk populations.

## 1. Introduction

Dementia and stroke are major neurological diseases and the leading causes of mortality in older adults (1–3). Our understanding of the genetic, lifestyle, and medical risk factors for these disorders has increased significantly (4). However, most of these studies have focused on individuals of white European descent (5, 6), while dementia and stroke are a global crisis affecting aging populations and societies worldwide. Moreover, the number of non-white citizens in Europe and the world has increased faster than the white population. It is essential to include underrepresented ethno-racially diverse groups in research on dementia and stroke (7–9) as there is a significant knowledge gap in the genetic epidemiology of non-whites, particularly in European countries. This gap concerns the incidence, genetic determinants, lifestyle risk factors, and co-morbidity across ethnic groups (10–12).

There are significant differences in morbidity across ethnicities, such as a higher prevalence of hypertension (13) and dyslipidemia (14) in Africans and diabetes in Asians, compared to Whites (15). Genetic analysis has become a major tool in the study of chronic and non-communicable diseases, as highlighted by the COVID-19 pandemic (16–20). Risk prediction typically includes risk factors such as age, sex, family history of the disease, and lifestyle (e.g., tobacco and alcohol consumption, physical activity) (21); however, in recent years, there has been increasing interest regarding including genomic information into risk models (22, 23). Polygenic risk scores (PRS) have been developed over the last two decades (24), and may lead to significant improvement in the prevention and management of diseases (e.g., selection of patient according to APOE status) (2).

Prediction of the risk of a disease is an essential part of preventative medicine, often guiding clinical management. PRS aggregate the effects of many genetic variants across the human genome into a single score and has recently been shown to have predictive value for multiple common diseases (25). However, most of these works were done in those of European descent and little is known about other ethnicities. Current studies using well-powered genome-wide association studies (GWAS) to assess the predictive value of PRS across a range of traits and populations have made a consistent observation: PRS predict individual risk far more accurately in Europeans than non-Europeans (26). Rather than chance or biology, this is a predictable consequence of the fact that the genetic discovery efforts to date heavily underrepresent non-European populations globally (6).

This study aims to quantify the risk of dementia, stroke, and mortality stratified by ethnicity and identify the factors driving these differences.

## 2. Methods

### 2.1. Data sources

This study is a part of the UK Biobank project 54,520. Complete descriptions of the UK Biobank have been presented elsewhere (27). Briefly, the UK Biobank is a large-scale population-based cohort study, including 500,000 subjects aged from 37 to 73 years during recruitment. The UK Biobank has approval from the North West Multi-center Research Ethics Committee (28). All included participants have signed the information consent form. All methods were carried out in accordance with relevant guidelines and regulations.

### 2.2. Clinical outcome and study variables

Participants with any prevalent dementia or stroke at baseline or younger than 55 years were excluded from their respective analysis, and participants without complete information about age, sex, and qualified genotype were also excluded. The population was divided into different ethnicities based on the touchscreen questionnaire at baseline. We studied dementia, stroke and mortality in Whites, Blacks, and Asians. The flow of the study participants is presented in Supplementary Figure 1. The clinical outcomes are (1) all-cause dementia, (29) including Alzheimer’s disease (AD), vascular dementia, and a part of unspecified dementia (2) stroke, (30) including ischemic and hemorrhagic stroke, and (3) mortality. The diseases were based on the self-reported illness from the verbal interview at baseline, or the ICD codes from hospital admission electronic health records in the primary or any secondary causes and/or death register. A Bonferroni correction was applied for the effective number of independent tests.

Risk factors of dementia previously identified (4) were used as explicative variables including low education, hearing loss, head injury, hypertension, alcohol assumption, obesity, smoking, major depression, social isolation, physical inactivity, diabetes, and air pollution using PM2.5. Other potential risk factors of dementia, i.e., age, sex, together with a family history of dementia, *APOE* genotype, and genetic risk score of dementia, were also of interest. For stroke and mortality analysis, atrial fibrillation was added in the risk factors based on the criteria of the revised Framingham Stroke Risk Profile (5). The definitions of the variables and the thresholds used are presented in Supplementary Table 1.

### 2.3. Genetic variants

UK Biobank genotyping was conducted by Affymetrix using the BiLEVEL Axiom array for ~50,000 participants and the remaining ~450,000 on the Affymetrix UK Biobank Axiom array (31). Detailed information on the genotyping process and technical methods are available online. We followed the UK Biobank’s recommendation to exclude the participants who had failed quality control, significant missing data or heterozygosity.

Genetic risk score (GRS) was explored in the current study. Thirty independent genetic determinants were selected from previous genome-wide association studies of AD in non-UK Biobank European populations (32–34). Their risk alleles and effect estimates on AD were extracted from the largest GWAS summary statistics (stage I) by Kunkle et al. which UK Biobank was not included (Supplementary Table 2) (35). Considering the disparity effect between *APOE* and other common variants, GRS without *APOE* variant was created for further analysis. The stroke data of the largest GWAS was used to create the GRS.

### 2.4. Statistical analysis

Cumulative incidence risk was calculated based on all incident cases occurring during the follow-up of the individuals without dementia and stroke at baseline using the cumulative incidence function (package etm) (36). Patients were censored at the date of the disease diagnosis, death, or the administrative censoring date, whichever came first. Mortality was accounted for as a competing event. The function estimates overall survival irrespective of cause of death by a modification of the Kaplan–Meier estimate, adapted for left truncation, and calculates age and cause-specific risk estimates and corresponding 95% CIs for the different ethnicities.

We compared the allele frequency of variants in Asian and black participants with the referent ethnicity, whites, by chi-square test. Hierarchical cluster analysis was used in the clustering analysis. Significance level corrected for the false discovery rate was considered.

All analyses were done in R (version 3.6.2) and the level of significance was set at *p* < 0.05 after Bonferroni’s correction.

## 3. Results

### 3.1. Prevalence of risk factors of dementia, stroke, and mortality across ethnicities

After excluding the participants younger than 55 years old, with prevalent dementia or stroke, or missing age, sex or genotype values at baseline, 272,660 participants were included in the final analysis, among whom the vast majority are White (97.80%), followed by Asian (1.35%), and black (0.84%). The mean duration of the follow-up was 11.2 years, yielding 3,050,595 person-years in total. Baseline characteristics of included participants are presented in Table 1. On average, white were older than Asian and black participants. Of note is that the genetic risk score for AD, the GRS was on average lower in black participants, yet twice as many individuals carry the rare high-risk APOE*44 genotype in black (5.1%) compared to white (2.3%) and Asians (1.1%; Supplementary Figure 2). On the other hand, the GRS for stroke was higher in black compared to white and Asian participants. White are almost twice as likely to have a family history of dementia compared to the other groups (14.9% for white, 6.0% for Asian and 8.2% for black).

**Table 1:** Characteristics of the participants at the inclusion, mean (standard deviation) and number (%) according to the type of variables.

	Variables	Whites (*n* = 266,671)	Asians (3,790)	Blacks (2,358)	*P*-value
Demographics and genetics	Age	62.5 (3.81)	62.1 (4.05)^***^	62.2 (4.14)^***^	**<0.001**
	Sex				
	Male	123,436 (46.3)	1,995 (54.4)	979 (42.5)	**<0.001**
	APOE				
	E22	1,668 (0.6)	6 (0.2)	24 (1.1)	**<0.001**
	E23	33,086 (12.4)	320 (8.7)	353 (15.2)	
	E24	6,639 (2.5)	32 (0.9)	158 (6.8)	
	E33	156,376 (58.6)	2,709 (73.3)	998 (43.5)	
	E34	62,690 (23.3)	577 (15.7)	646 (28.1)	
	E44	6,212 (2.3)	42 (1.1)	124 (5.4)	
	PRS				
	Dementia	4.45 (0.53)	4.43 (0.49)	4.10 (0.42)^***^^^^	**<0.001**
	Stroke	1.27 (0.23)	1.30 (0.25)	1.39 (0.28) ^***^^^^	
	Family history of dementia				
	Yes	39,934 (15.0)	223 (6.0)	189 (8.2)	**<0.001**
Lifestyle	Education				
	Low education	186,654 (70.7)	2,072 (56.2)	1,667 (72.4)	**<0.001**
	Alcohol consumption				
	Never	10,154 (3.8)	1,331 (36.1)	361 (15.7)	**<0.001**
	Previous	9,675 (3.6)	222(6.1)	150 (6.5)	
	Current	246,618 (92.5)	2,099 (56.9)	1,777 (77.2)	
	Body Mass Index	27.6 (4.6)	27.3 (4.2)^***^	29.7	**<0.001**
	Obesity	67,518 (24.8)	806 (21.3)	(5.1)^***^^^^961 (40.8)	**<0.001**
	Tobacco				
	Never	134,445 (50.4)	2,798 (75.9)	1,662 (72.2)	**<0.001**
	Previous	108,156 (40.1)	607 (16.5)	438 (19.0)	
	Current	22,923 (8.6)	240 (6.5)	177 (7.7)	
	Physical inactivity				
	Yes	37,977 (14.2)	646 (17.5)	320 (13.9)	**<0.001**
	Social isolation				
	Yes	76,756 (28.8)	1,178 (32.0)	523 (22.7)	**<0.001**
	PM2.5	9.91 (1.03)	10.30 (0.96)^***^	10.70 (1.05)^***^^^^	**<0.001**
Comorbidities	Hearing loss				
	Yes	78,498 (29.4)	849 (23.0)	333 (14.5)	**<0.001**
	Prevalent head injury				
	Yes	3,191 (0.1)	45 (0.1)	18 (0.08)	**<0.001**
	Prevalent hypertension	166,430 (62.4)	2,562 (69.5)	1,824 (79.2)	**<0.001**
					
	Major Depression				
	Yes	19,744 (7.4)	193 (5.2)	96 (4.2)	**<0.001**
	Prevalent diabetes				
	Yes	18,377 (6.9)	1,060 (28.8)	524 (22.8)	**<0.001**
	Prevalent atrial fibrillation				
	Yes	2,826 (1.1)	8 (0.2)	8 (0.3)	**<0.001**

^*^p < 0.05, ^**^p < 0.01, ^***^p < 0.001 difference with White (Bonferroni correction).

^^^*p* < 0.05, ^^^^*p* < 0.01, ^^^^^*p* < 0.001 difference Asian and Black (Bonferroni correction).

The *p*-values are the results of the ANOVA or chi^2^ test.Bold values indicate significant results.

**Figure 1:**
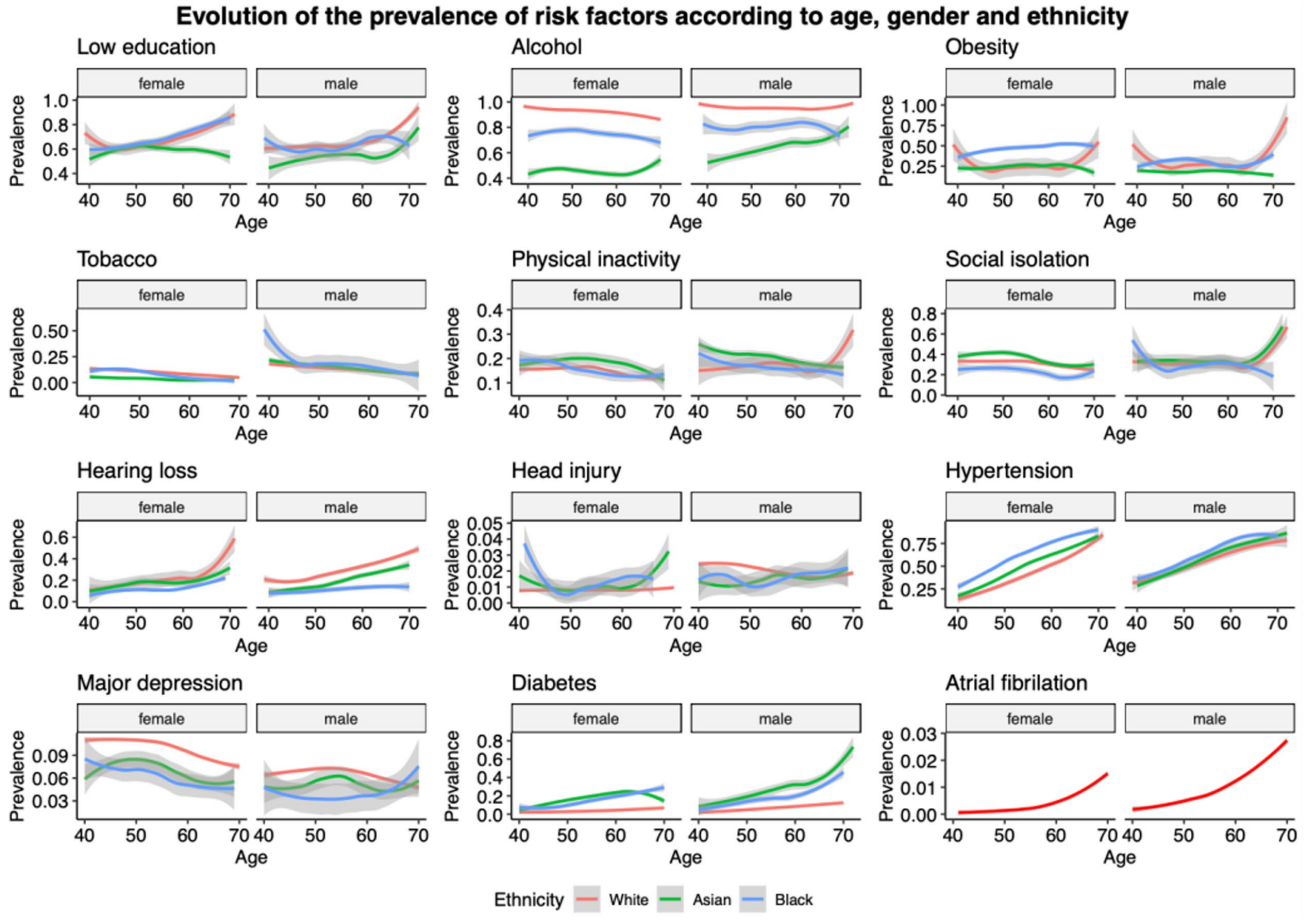
Repartitions of lifestyle and comorbidities by sex across ages for the different ethnicities. For atrial fibrillation due to the low number of cases in black (*n =* 8) and Asian (*n =* 8), only white participants’ results are presented.

Figure 1 shows the breakdown of lifestyle factors and comorbidities by sex across age groups by ethnicity. Of note is that across age, the proportion of people with low education is similar in white (70.7) and black (72.4) participants but lower in Asian (56.2). Alcohol consumption is most frequent in white people compared with other ethnicities. The proportion of obesity is highest in black, and in particular women, while the proportion of physical inactivity is highest in Asian. Hypertension and diabetes are more prevalent in Asian people (70.0% for hypertension and 29.4% for diabetes) and black participants (79.4% for hypertension and 23.1% for diabetes) compared to white (62.8% for hypertension and 7.1% for diabetes). Of note is that white (29.6%) and Asian (23.3%) suffered more often from hearing loss than black participants (14.4%) across age. Atrial fibrillation is more frequent in white (1.1%) compared to Asian (0.2%) and black (0.3%; see Table 1 for complete results).

**Figure 2:**
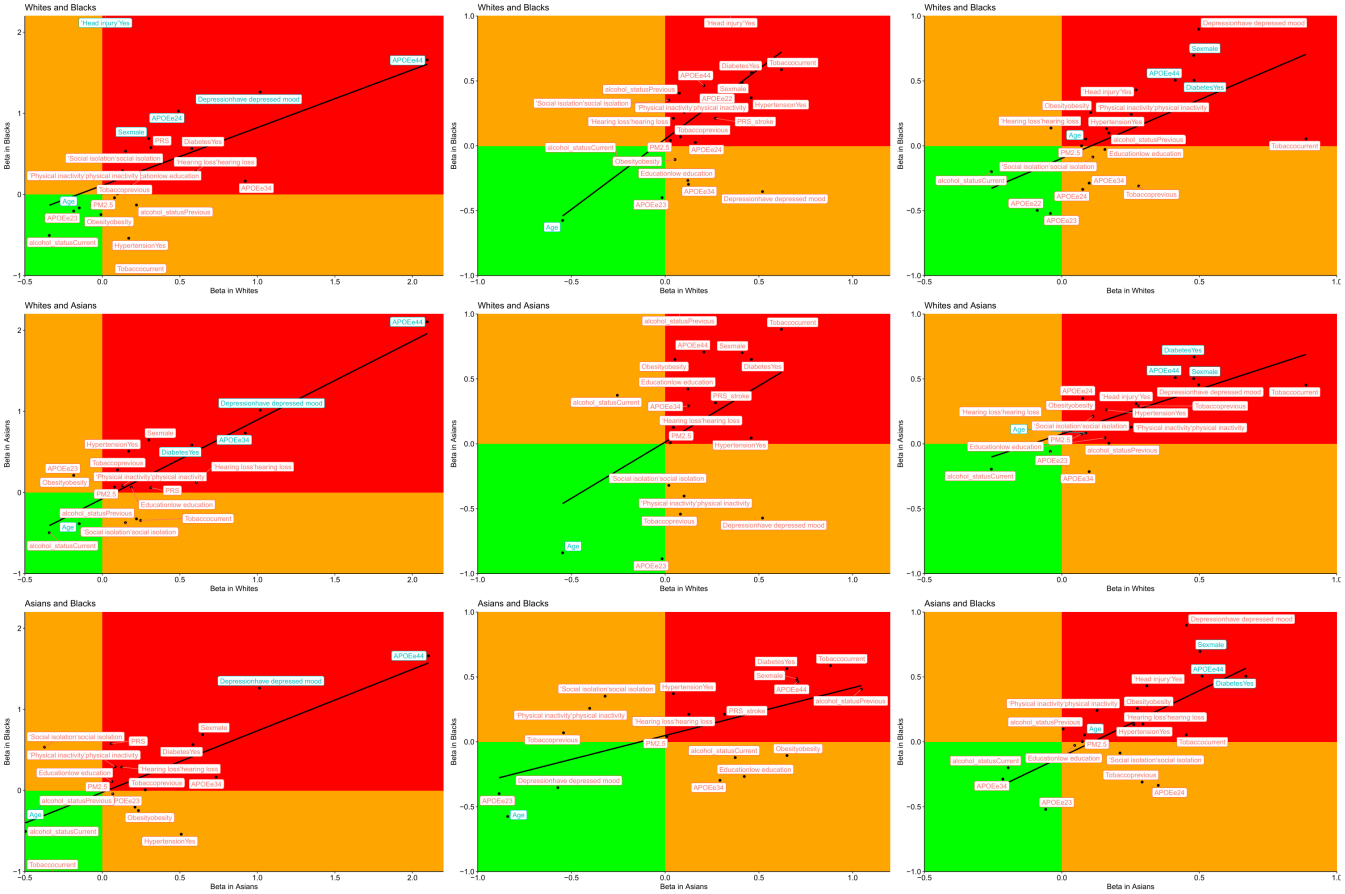
Correlations between risk factors of dementia (left columns), stroke (middle columns) and mortality (right columns) by ethnicity, blue colour indicates the variables found to be significantly different between the two ethnicities.

Table 2 and Supplementary Figure 3 give a global overview of the risk estimates for each risk factor previously (4, 5) across different ethnicities for dementia, stroke and mortality. To further compare the ethnicities, we plotted the beta of the different risk factors in Figure 2. Below we discussed the overall findings for dementia, stroke, and mortality.

**Table 2:** Results of the multivariate cox-regression.

Variables	Dementia			Stroke			Mortality		
	Whites (4,603/266,671)	Asians (68/3,790)	Blacks (71/2,358)	Whites (6,503/266,671)	Asians (95/3,790)	Blacks (82/2,358)	Whites (23,216/266,671)	Asians (280/3,790)	Blacks (169/2,358)
Age	1.22 [1.21–1.23]^***^	1.13 [1.04–1.22]^**^	1.21 [1.15–1.30]^***^	0.58 [0.57–0.59]^***^	0.43 [0.35–0.53]^***^	0.56 [0.48–0.66]^***^	1.09 [1.08–1.09]^***^	1.09 [1.04–1.13]^***^	1.05 [1.01–1.10]^*^
Sex (Male)	1.36 [1.26–1.46]^***^	1.86 [0.90–3.83]	1.95 [1.03–3.73]^*^	1.51 [1.41–1.61]^***^	2.02 [1.01–4.08]^*^	1.62 [0.89–2.95]	1.61 [1.56–1.67]^***^	1.65 [1.13–2.40]^**^	2.00 [1.35–2.99]^***^
*APOE*									
*E33*	Ref			Ref			Ref		
*E22*	1.11 [0.64–1.92]	**/**	**/**	1.22 [0.86–1.74]	**/**	1.39 [0.18–10.65]	0.91 [0.73–1.13]	**/**	0.61 [0.08–4.43]
*E23*	0.83 [0.72–0.96]^*^	**/**	0.85 [0.28–2.57]	0.98 [0.89–1.08]	0.41 [0.09–1.73]	0.67 [0.27–1.65]	0.96 [0.91–1.01]	0.94 [0.55–1.62]	0.59 [0.30–1.17]
*E24*	1.59 [1.27–1.99]^***^	1.24 [0.43–3.56]	2.76 [1.05–7.52]^*^	1.17 [0.97–1.40]	**/**	1.03 [0.34–3.04]	1.08 [0.97–1.19]	1.42 [0.34–5.82]	0.71 [0.30–1.68]
*E34*	2.44 [2.25–2.64]^***^	2.07 [1.04–4.13]^*^	1.20 [0.57–2.53]	1.13 [1.05–1.21]^***^	1.34 [0.69–2.59]	0.74 [0.38–1.43]	1.10 [1.06–1.14]^***^	0.81 [0.51–1.27]	0.75 [0.48–1.16]
*E44*	7.66 [6.80–8.62]^***^	9.83 [2.89–33.48]^***^	5.19 [2.11–12.73]^***^	1.22 [1.01–1.48]^*^	2.02 [0.26–15.46]	1.59 [0.53–4.68]	1.51 [1.38–1.65]^***^	1.66 [0.68–4.14]	1.66 [0.83–3.29]
PRS	1.36 [1.27–1.45]^***^	1.14 [0.63–2.05]	1.81 [0.91–3.58]	1.31 [1.15–1.48]^***^	1.37 [0.46–4.04]	1.26 [0.46–3.42]	/	/	/
Family history (dementia)	1.35 [1.24–1.47]^***^	1.25 [0.38–4.08]	0.90 [0.31–2.62]	/	/	/	/	/	/
Low education	1.21 [1.11–1.32]^***^	1.09 [0.60–2.00]	1.09 [0.54–2.28]	1.13 [1.05–1.21]^***^	1.52 [0.85–2.59]	0.76 [0.41–1.42]	1.16 [1.12–1.21]^***^	1.05 [0.77–1.43]	0.97 [0.63–1.49]
Alcohol consumption									
Never	Ref			Ref			Ref		
Previous	1.24 [1.01–1.53]^*^	0.68 [0.19–2.37]	0.95 [0.28–3.25]	1.08 [0.88–1.31]	2.84 [0.95–8.48]	1.50 [0.51–4.42]	1.18 [1.07–1.31]^***^	1.00 [0.54–1.83]	1.10 [0.53–2.79]
Current	0.71 [0.60–0.83]^***^	0.59 [0.30–1.16]	0.62 [0.28–1.36]	0.77 [0.67–0.90]^***^	1.43 [0.71–2.97]	0.88 [0.39–1.96]	0.77 [0.71–0.84]^***^	0.82 [0.58–1.18]	0.82 [0.48–1.38]
Obesity	0.98 [0.90–1.07]	1.30 [0.65–2.61]	0.83 [0.44–1.55]	1.05 [0.99–1.13]	1.91 [0.99–3.69]	0.89 [0.51–1.60]	1.11 [1.07–1.15]^***^	1.31 [0.92–1.89]	1.29 [0.88–1.89]
Tobacco									
Never	Ref			Ref			Ref		
Previous	1.13 [1.04–1.22]^**^	1.26 [0.61–2.61]	0.95 [0.27–3.25]	1.08 [1.01–1.16]^*^	0.58 [0.26–1.31]	1.07 [0.52–2.18]	1.32 [1.27–1.37]^***^	1.34 [0.92–1.95]	0.73 [0.44–1.22]
Current	1.29 [1.13–1.47]^***^	0.65 [0.15–2.81]	0.62 [0.28–1.36]	1.85 [1.69–2.04]^***^	2.41 [1.04–5.58]^*^	1.79 [0.71–4.51]	2.42 [2.31–2.54]^***^	1.57 [0.88–2.78]	1.05 [0.55–2.03]
Physical inactivity	1.15 [1.05–1.26]^***^	1.09 [0.55–2.16]	1.39 [0.70–2.75]	1.11 [1.02–1.19]^**^	0.68 [0.32–1.31]	1.29 [0.69–2.42]	1.28 [1.23–1.33]^***^	1.14 [0.79–1.62]	1.27 [0.83–1.96]
Social isolation	1.14 [1.06–1.24]^***^	0.70 [0.35–1.40]	1.63 [0.85–3.14]	1.02 [0.95–1.09]	0.73 [0.38–1.37]	1.42 [0.77–2.63]	1.12 [1.08–1.16]^***^	1.23 [0.89–1.70]	0.92 [0.57–1.47]
PM2.5	1.08 [1.05–1.12]^***^	1.11 [0.83–1.48]	0.95 [0.71–1.27]	1.03 [0.99–1.06]	1.01 [0.76–1.33]	1.04 [0.81–1.33]	1.07 [1.06–1.09]^***^	1.08 [0.93–1.25]	1.00 [0.84–1.19]
Hearing loss	1.02 [0.94–1.10]	1.16 [0.61–2.19]	1.35 [0.67–2.72]	1.04 [0.98–1.11]	1.13 [0.60–2.12]	1.23 [0.63–2.42]	0.96 [0.93–0.99]^*^	1.34 [0.97–1.85]	1.14 [0.71–1.85]
Head injury	1.83 [1.47–2.27]^***^	/	20.03 [5.55–74.32]^***^	1.41 [1.15–1.74]^**^	**/**	13.96 [3.14–62.12]^***^	1.31 [1.17–1.43]^***^	1.36 [0.43–4.31]	1.54 [0.37–6.40]
Hypertension	1.20 [1.10–1.31]^***^	1.68 [0.74–3.84]	0.57 [0.27–1.21]	1.58 [1.47–1.70]^***^	1.04 [0.53–2.04]	1.44 [0.64–3.28]	1.17 [1.13–1.22]^***^	1.30 [0.88–1.92]	1.14 [0.68–1.90]
Major depression	2.78 [2.53–3.06]^***^	2.78 [1.05–7.29]^*^	3.42 [1.35–8.68]^**^	1.68 [1.53–1.85]^***^	0.56 [0.07–4.16]	0.70 [0.16–2.96]	1.64 [1.56–1.73]^***^	1.57 [0.93–2.65]	2.45 [1.35–4.34]^**^
Diabetes	1.78 [1.59–1.96]^***^	1.83 [1.00–3.38]^*^	1.72 [0.94–3.17]	1.58 [1.45–1.74]^***^	1.91 [1.08–3.39]^*^	1.75 [0.98–3.13]	1.62 [1.54–1.69]^***^	1.95 [1.42–2.68]^***^	1.66 [1.13–2.43]^*^
Atrial fibrillation	1.16 [0.89–1.51]	/	/	1.91 [1.59–2.30]^***^	**/**	/	1.39 [1.24–1.56]^***^	3.55 [1.09–11.38]^*^	**/**

^*^p < 0.05, ^**^p < 0.01, ^***^p < 0.001.

Results are presented as hazard ratio and 95% confidence intervals.Bold values indicate significant results.

### 3.2. Risk of dementia by ethnicity

**Figure 3:**
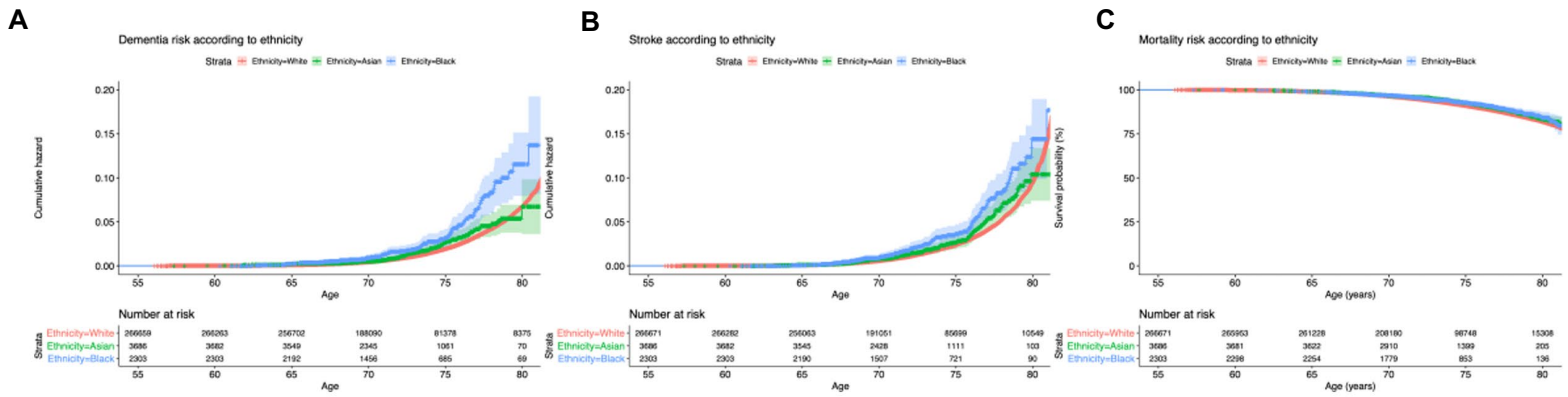
Survival curves of the risk of dementia **(A)**, stroke **(B)** and mortality **(C)**, according to age and ethnicity. Red color is for White, green for Asian and blue for black. Colored area represents the 95%CI.

**Figure 4:**
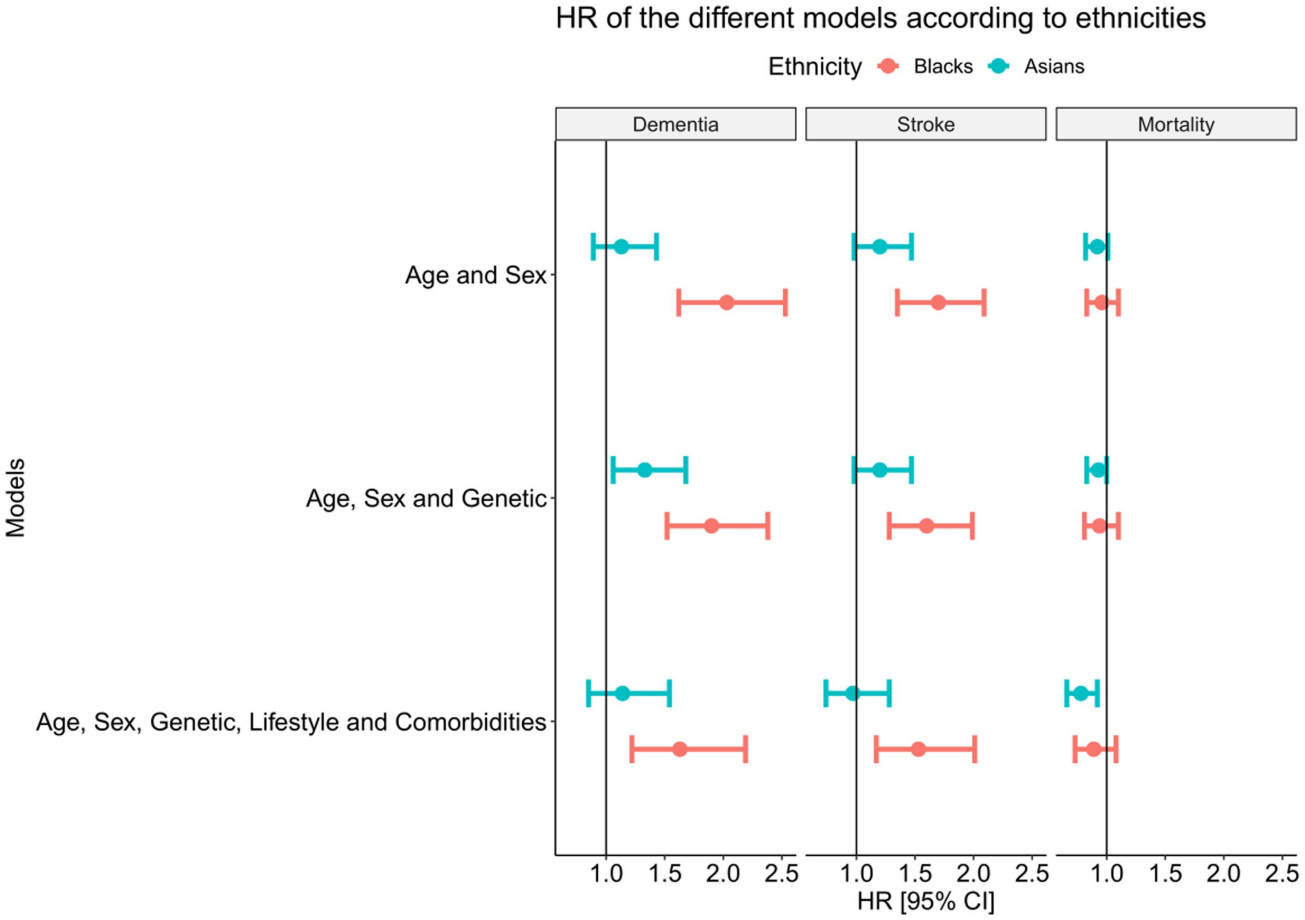
Hazard ratios and 95%CIs for the risk of dementia, stroke and mortality according to ethnicity. Whites are used as the reference group.

During follow-up, 4,742 participants developed dementia. The incidence of dementia is 160 cases/100,000 person-years in white, 178 cases/100,000 person-years in Asian, and 274/100,000 person-years in black. Figure 3A shows the cumulative incidence of dementia by age across ethnicities. This increased risk in black participants is significantly different from that of white (*p* = 0.003). Controlling for age and sex, we found an increased risk of dementia for black relative to white [Figure 4, HR = 2.03 (95%CI 1.62–2.53), *p* < 0.001] but not for Asian (HR = 1.13 [0.89–1.43], *p* = 0.288). Figure 4 shows that after adjusting further for *APOE* and the GRS of AD, the difference is still significant for black (HR = 1.90 [1.52–2.38], *p* < 0.001) and Asian participants (HR = 1.33 [1.06–1.68], *p* = 0.015). Finally, when further adjusting for all risk factors, including lifestyle factors and comorbidities, the HR in black decreased but remained significant (HR = 1.63 [1.22–2.19], *p* = 0.002), suggesting that differences in lifestyle and comorbidity explain part but not all of the increase in risk.

**Figure 5:**
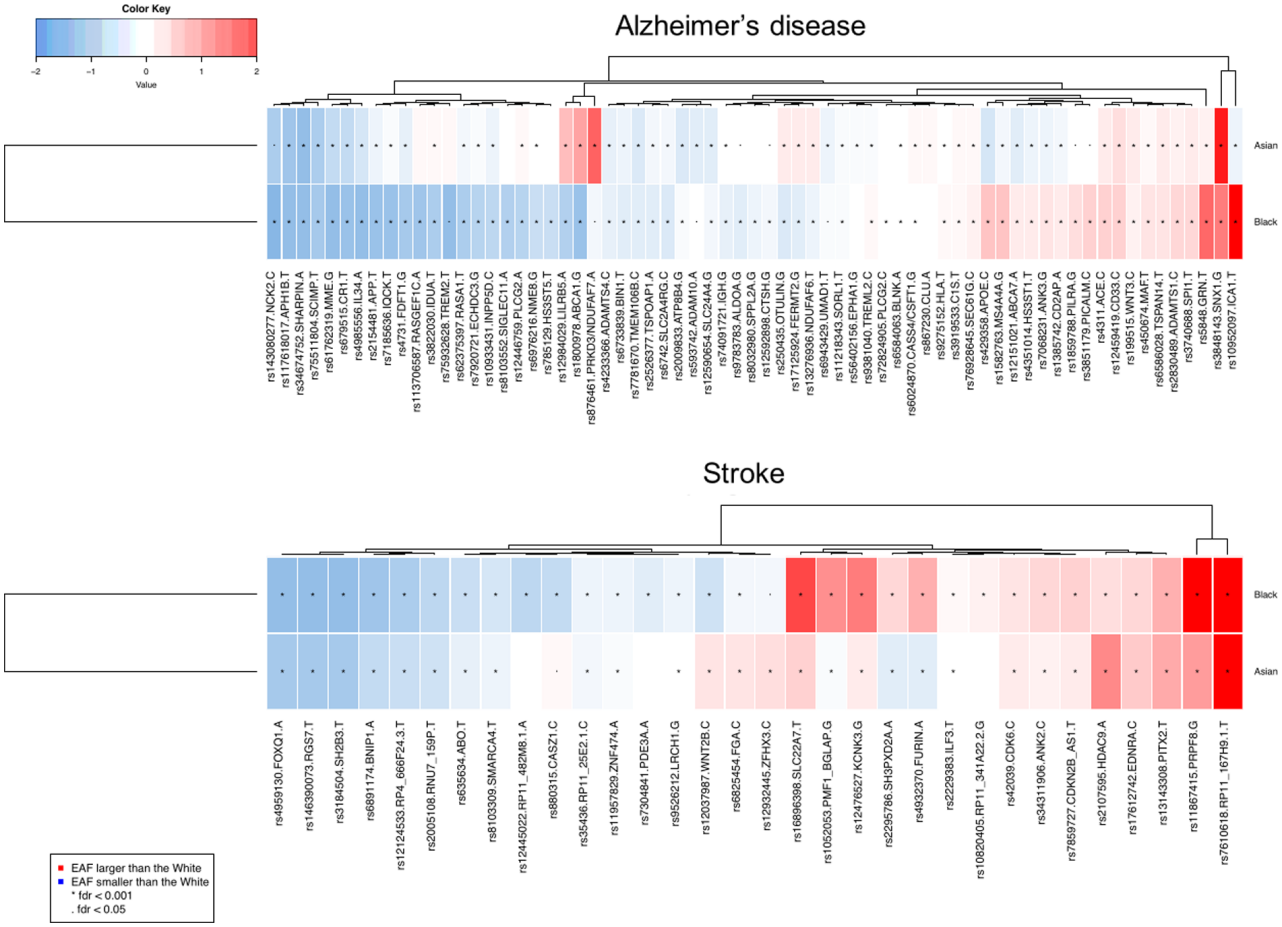
The deviation of referent allele frequency of Asians and Blacks compared with the whites in the AD variants reported in previous GWAS studies (Chi-square test was performed to identify the statistical difference between frequencies). Red: Risk allele frequency larger than White population; blue: referent allele frequency smaller than White population. The depth of color range from +/− 2 to zero. ^*^FDR < 0.001, FDR < 0.05.

For Asians, the same trend is seen but the HR is not significantly increased compared to white [Figure 4, HR = 1.14 (0.85–1.54), *p* = 0.205] when adjusting for lifestyle and comorbidity, suggesting these factors explain most of the differences. Most of the morbidity and lifestyle effect is explained by physical activity and diabetes (Figure 1 and Table 2). Finally, to understand the difference of the GRS of AD in black and white participants (Table 1), we examined the differences in the allele frequency of AD SNPs across ethnicities (Figure 5). Compared to the white population, more than half of the SNPs (63%) in the black population have a significantly lower frequency of AD risk variants than in whites. The differences between white and Asian (55%) is not as strong as the differences between white and black.

### 3.3. Risk of stroke by ethnicity

A total of 6,680 participants had at least one stroke during follow-up. The incidence of stroke is 203/100,000 person-year in white, 274/100,000 person-years in Asian, and 234/100,000 person-years in black. Figure 3B shows a significant difference in the incidence of stroke among ethnicities (*p* < 0.001). White had a slightly older age at stroke onset: 72.7 ± 4.3 years compared to 72.3 ± 4.4 in Asian (*p*_compared to whites_ < 0.001) and 72.2 ± 4.6 in Black people (*p*_compared to whites_ < 0.001). Black participants had an increased risk of stroke relative to white participants (Figure 4, HR = 1.70 [1.35–2.09], *p* < 0.001—when adjusting for age and sex, HR = 1.60 [1.28–1.99], *p* < 0.001—when further adjusting for genetic risk factors, HR = 1.53 [1.17–2.01], *p* < 0.001—when further adjusting for lifestyle and comorbidities). Interestingly, the change in the hazard ratio of stroke is less pronounced than that of dementia. The risk of stroke is not different in Asian compared to white [HR =0.97 [0.74–1.28], *p* = 0.925] when adjusting for age, sex, genetic risk factors, lifestyle, and comorbidities. Concerning the genetic difference, less than half of the SNPs (53%) in the black population have a significantly lower frequency of stroke risk variants than in white participants.

### 3.4. Risk of mortality By ethnicity

Concerning mortality, 23,665 people died during the follow-up. The incidence of mortality is 801/100,000 person-years in white, 727/100,000 person-years in Asian, and 716/100,000 in black people. There are no statistical differences in the age of death among the ethnicities (*p* = 0.65, Figure 3C). Figure 4 shows that the risk of death is not different in black compared to white participants (HR = 0.89 [0.73–1.08], *p* = 0.095) while the risk of mortality significantly decreased in Asian participants (HR = 0.78 [0.66–0.92], *p* = 0.034) when adjusting for age, sex, genetic, lifestyle and comorbidities.

## 4. Discussion

Within the UK Biobank, we find those who identified themselves as black participants are at increased risk of dementia and stroke. The increased risk cannot be explained by our current knowledge of risk factors. Adjusting for genetic factors, lifestyle, and comorbidity, the risk of dementia in Asian is similar to that in white participants, while the risk of stroke is similar to that in white people. We do not find a difference in mortality in black compared to white participants. In Asian, mortality is significantly less likely than in white after accounting for adjustment lifestyle and morbidity factors.

The main finding of this study is the high risk of dementia and stroke in Black people participating in the UK Biobank compared to Whites and Asians. These results are in line with the results of large observational studies in the United States of subjects older than 65 where African Americans have the highest prevalence of dementia (14.7%), followed by Hispanics (12.9%), and non-Hispanic whites (11.3%) (37). As expected, black UK Biobank participants presented more comorbidities associated with dementia and stroke (i.e., obesity, diabetes, hypertension) (4) and are subject to higher levels of air pollution compared to white. Of note is that the level of education is similar for white and black UK Biobank participants and the risk of mortality is not increased in black compared to white people. These findings do not exclude inequalities between white and black participants, e.g., schooling and healthcare infrastructure in the general population. However, in the setting of the UK Biobank, it is unlikely that these inequalities explain the higher risk of dementia in black. As we find that most risk factors have similar associations for the two ethnicities, the differences in the effect and frequencies of *APOE* may be relevant and raises the question to what extent the observed risk difference is explained by genetic factors. Of note is that despite the small sample size, we find an unfavorable distribution of *APOE* genotypes in black. By contrast, the GRS values are significantly lower in black compared to white participants which is a protective factor (38). Though the proportion of APOE*44 carriers is higher in Black (5.4%) than in the white population (2.3%, *p* < 0.001, Table 1), the effect of the APOE*44 variant on the risk of incident dementia in the Black population is much smaller than in white population, suggesting there are modifying variants in people of African descent in the UK, as has been found in African Americans (39). Although this may seem at odds with the finding that a family history of dementia does not increase the risk of dementia in black and Asian participants, one may argue that family history in immigrants is less reliable compared to white people (40). Similarly, we find that black participants have an increased risk of stroke compared to white that is not explained by the genetic factors, lifestyle, and morbidity known to be involved in stroke incidence (41). Also for stroke, we found differences in allele frequencies (42).

Compared to white participants, Asian do not have a significantly increased risk of dementia nor stroke when we adjust for genetic factors (43), lifestyles, and morbidity (12, 44, 45). Although the risk of dementia is increased in Asian compared to white people, this is in large part explained by lifestyle and morbidity (15). The risk of stroke in Asian is not significantly increased and is very similar to that of white after adjustment for additional lifestyle and comorbidity factors (46).

The distribution of *APOE* in Asian is also different from the white population with a higher proportion of APOE*33 carriers and fewer APOE*34 and APOE*44 carriers. GRS score in this group however, does not differ from the white study population. A recent study suggests that genetic factors found predominantly through European-GWASs may play a limited role in South Asians (45).

For dementia, we found the PRS (excluding *APOE*) in Black participants is lower than in Whites. A higher frequency of APOE*44 in black compared to white and Asian participants is fund, but the effect estimates of *APOE* on incident dementia is much lower in black than in white. Differences in frequencies and effect have also been reported for *ABCA7* (47). These inconsistencies suggest that the PRS calculated based on the SNPs identified from the GWAS of white population may be generalizable to other populations (48). We further find that the allele frequency of most of the SNPs included in the GRS are significantly different among ethnicities, especially between black and white populations. This finding highlights the importance of generating large GWAS of dementia in the African population and that unique genetic loci associated with dementia are highly expected to emerge in such studies. In agreement with various studies in the United States, we identified that one-third of the SNPs, which have been previously found to explain the differences of risk of AD between ethnicities, are in the different direction as APOE*4 SNPs. This is to say, the two *APOE* SNPs have increased frequency in black people, but many other genetic variants surrounding the APOE*2/3/4 variants differ between white and black participants. These genetic variants may modify the effects seen in black and white. Similar findings are shown in Asian as well, which was not as strong as in black. It is of interest that we and others find this for dementia genes but not genetic variants implicated in stroke (49). On the other hand for stroke, we find its PRS for black participants is higher which explained the overall increased risk (50).

Compared with earlier observational studies (37, 51), the strength of this study is that we adjusted our analysis for identified lifestyle and genetic risk factors. Despite those adjustments, we still found an increased risk of dementia in black people (HR = 1.60 [1.19–2.14]). Concerning the effect of APOE*4, our results are in accordance with a follow-up study of 1,871 African Americans, of whom 182 developed dementia, the authors found an HR of 4.12 [2.33–7.28] for APOE*44 carriers compared to APEOE*33; (52) which is similar to or results of 4.66 [2.01–10.81]. Interestingly the patterns of linkage between the ε4 allele of *APOE* and `523 poly-T alleles in the adjacent gene, *TOMM40*, differ between white and African Americans, both genotypic and allelic data support that among African Americans the ε4-`523-L haplotype had a stronger effect on the risk of AD than other ε4-`523 haplotypes (53).

This study has a few limitations. The most important one is the unbalanced make up of ethnicities in UK Biobank relative to the general population. Another limitation is the relatively young age of the participants at the inclusion (62.5 ± 3.8 years old) and the relatively short duration of follow-up (11.2 ± 1.8 years). These two factors imply that there have been few cases of incident cases and therefore a reduction in study power. Another limitation is that socioeconomic levels were not included in our analysis while we know this is an important factors of dementia and that there are huge disparities across ethnicities (54). Selection bias is also a concern. Black participants in our study sample have a similar educational attainment to white participants, which demographic studies suggest is not the case in the UK general population (55). This would imply that black and white in our study are more similar in dementia, stroke, and mortality risk than actually is the case in the general population. Thus, the increase in risk seen in black participants for dementia or stroke, is likely an underestimate. Finally, it has been shown that UK Biobank’s participants are not representative of the population, with evidence of a ‘healthy volunteer’ selection bias. Nonetheless, the valid assessment of exposure-disease relationships may be widely generalizable and does not require participants to be representative of the population at large (56).

## 5. Conclusion

An important finding of our study is that there are no major differences in mortality across ethnicities among UK Biobank participants that may bias the risk estimates for stroke and dementia. This study emphasizes the need for more heterogeneity in large scale hypothesis-free cohort studies to understand the differences in risk of major diseases such as dementia and stroke and how this relates to genetic, lifestyle, and morbidity factors. The inclusion of participants of different ethnic backgrounds will increase the available statistical power and could lead to more targeted prevention campaigns. This same argument can be made for clinical trials (57, 58). This research is key for the future prevention of dementia and stroke in low and mid-income countries (59). With the emergence of gene therapy and precision medicine, the question of health inequalities related to genetic and epidemiologic research becomes increasingly urgent. To close this gap in our knowledge we need major investments in ethnically diverse biobanks in the UK and elsewhere.

## Data availability statement

Publicly available datasets were analyzed in this study. This data can be found here: UK Biobank.

## Ethics statement

The studies involving human participants were reviewed and approved by North West Multi-center Research Ethics Committee. The patients/participants provided their written informed consent to participate in this study.

## Author contributions

The study was conceived by BB and CD. BB and JL performed the statistical analysis. NA and CD verified the analytical methods. BB, JL, AT, NA, and CD did the data interpretation. CD supervised the findings of this work. All authors contributed to the article and approved the submitted version.

## Funding

This research has been conducted using data from UK Biobank, a major biomedical database. The current study was authorized from UK Biobank project 54520. This project was funded through the King Abdulaziz University & Oxford University Centre for Artificial Intelligence in Precision Medicines (KO-CAIPM, CMR0020).

## Conflict of interest

The authors declare that the research was conducted in the absence of any commercial or financial relationships that could be construed as a potential conflict of interest.

## Publisher’s note

All claims expressed in this article are solely those of the authors and do not necessarily represent those of their affiliated organizations, or those of the publisher, the editors and the reviewers. Any product that may be evaluated in this article, or claim that may be made by its manufacturer, is not guaranteed or endorsed by the publisher.
