# Supplementary material for: Does ethnicity influence dementia, stroke and mortality risk? Evidence from the UK Biobank
Source: Front Public Health. 2023 Apr 14;11:1111321. doi: 10.3389/fpubh.2023.1111321 (PMC10140594; doi:10.3389/fpubh.2023.1111321)
Supplement: Supplementary file 1 [file Data_Sheet_1.docx]

Does ethnicity influence dementia, stroke and mortality risk? Evidence from the UK Biobank

Prof. Bruno Bonnechère^1,2,3*^, Dr. Jun Liu^1*^, Msc. Alexander Thomson^1^, Prof. Najaf Amin^1^, Prof. Cornelia van Duijn^1^

^1^  Nuffield Department of Population Health, University of Oxford, Oxford, United-Kingdom

^2^ REVAL Rehabilitation Research Center, Faculty of Rehabilitation Sciences, Hasselt University, Diepenbeek, Belgium

^3^ Technology-Supported and Data-Driven Rehabilitation, Data Science Institute, Hasselt University, Diepenbeek, Belgium

Supplementary material

**Supplementary Table 1: Risk factors definitions**

| **Domain** | **Risk factors** | **Definitions** | **Sources** |
| --- | --- | --- | --- |
| Lifestyle | **Education**  Low education | Participants without a college or university degree, or A levels or equivalent (academic advanced-levels, post-compulsory education) | UK Biobank Touchscreen questionnaire at baseline |
|  | **Alcohol consumption**  None  Low  Mid  High | less than one dose per week  1-2 per week  3–4 times per week  daily or almost daily | UK Biobank Touchscreen questionnaire at baseline |
|  | **Body Mass Index**  Obesity | >30 kg/m^2^ calculated from measured weight and height | UK Biobank Touchscreen questionnaire at baseline |
|  | **Tobacco**  Never  Previous  Current |  | UK Biobank Touchscreen questionnaire at baseline |
|  | **Physical inactivity**  Yes | Participants in the low IPAQ (International Physical Activity Questionnaire) group | UK Biobank Touchscreen questionnaire at baseline |
|  | **Social isolation**  Yes | Answer to the question “Which of the following do you attend once a week or more often?” was none (no group activity). | UK Biobank Touchscreen questionnaire at baseline |
| Comorbidities | **Hearing loss**  Yes | Answer to the question “Do you have any difficulty with your hearing?” is yes or completely deaf | UK Biobank Touchscreen questionnaire at baseline |
|  | **Prevalent head injury**  Yes | Participants that ever had neurological / intracranial injury or trauma and fracture of skull or head before the first assessment: self-reported neurological injury, trauma, fracture of skull or head, codes S00-S09 in ICD-10, and codes 800-804 or 850-854 in ICD-9. | UK Biobank Touchscreen questionnaire at baseline |
|  | **Prevalent hypertension**  Yes | Blood pressure medication taken from the touchscreen question “Do you regularly take any of the following medications?”, and/or the mean of twice automated reading measured blood pressure: systolic blood pressure not less than 140 mmHg, or diastolic blood pressure not less than 90 mmHg. | UK Biobank Touchscreen questionnaire at baseline |
|  | **Major Depression**  Yes | Participant that had self-reported single episode, recurrent moderate, recurrent severe, or with codes F32 (single episode) and/or F33 (recurrent) in ICD-10 by the first assessment. | UK Biobank Touchscreen questionnaire at baseline |
|  | **Prevalent diabetes**  Yes | 1) Answer to the question “Has a doctor ever told you that you have diabetes?” as yes; 2) self-reported diabetes, type 1 diabetes, type 2 diabetes, gestational diabetes 3) codes E10-E14 (diabetes mellitus) or O24 (diabetes mellitus in pregnancy) in ICD-10, and codes 250 (diabetes mellitus) or 2535 (diabetes insipidus) in ICD-9; 4) HbA1c ≥ 6.5% (48 mmol/mol) | UK Biobank Touchscreen questionnaire at baseline |
|  | **Prevalent cardiovascular disease**  Yes | Based on diseases of the circulatory system from self-reported illness, codes I00-I99 in ICD-10, or codes 390-459 in ICD-9 | UK Biobank Touchscreen questionnaire at baseline |
|  | **Prevalent stroke**  Yes | Based on the previous outcome adjudication guidelines in UK Biobank: from self-reported illness and/or ICD codes (I60, I61, I63, I64.X) | UK Biobank Touchscreen questionnaire at baseline |
|  | **Prevalent dementia**  Yes | Based on the previous outcome adjudication guidelines in UK Biobank: from self-reported illness and/or ICD codes (F00, F01, F02) | UK Biobank Touchscreen questionnaire at baseline |
|  | **Prevalent dyslipidemia**  Yes | Based on the cholesterol lowering medication taken from the question “Do you regularly take any of the following medications?”, and/or the National Cholesterol Education Program-Adult Treatment Panel III as either total cholesterol ≥ 240mg/dl, LDL-cholesterol ≥ 160 mg/dl, HDL-cholesterol < 40 mg/dl, or triglycerides ≥ 200 mg/dl | UK Biobank Touchscreen questionnaire at baseline |
|  | **Family history of dementia**  Yes | Based on the questionnaires if the participants had a biological father, mother and siblings who was diagnosed withAD/dementia | UK Biobank Touchscreen questionnaire at baseline |
|  | **Prevalent atrial fibrillation**  **Yes** | Based on the previous outcome adjudication guidelines in UK Biobank: from self-reported illness and/or ICD codes. | UK Biobank Touchscreen questionnaire at baseline |
| Ethnicity  White  Asian  African | | White British, Irish, or any other white ethnic background  Indian, Pakistani, Bangladeshi  Caribbean, African, or any other black ethnic background | UK Biobank Touchscreen questionnaire at baseline |

**Supplementary Table 2: SNPs included in the dementia genetic risk score (including APOE)**

| **SNP** | **Anotate (SNPid.gene or nearest genes.risk allele)** | **First resource (Author)** | **Chromosome (kunkle)** | **Position (Kunkle)** | **Effect allele (Kunkle)** | **Non Effect allele (Kunkle)** | **Beta (Kunkle)** | **SE (Kunkle)** | **Pvalue (Kunkle)** | **Effect allele freq (Kunkle)** |
| --- | --- | --- | --- | --- | --- | --- | --- | --- | --- | --- |
| rs6656401 | rs6656401.CR1.A | Lambert | 1 | 207692049 | A | G | 0·147 | 0·019 | 2·17E-15 | 0·188 |
| rs6733839 | rs6733839.BIN1/NIFKP9.T | Lambert | 2 | 127892810 | T | C | 0·169 | 0·015 | 4·02E-28 | 0·407 |
| rs35349669 | rs35349669.INPP5D.T | Lambert | 2 | 234068476 | T | C | 0·043 | 0·015 | 2·90E-03 | 0·48 |
| rs6448807 | rs6448807.RP11_281P23.2.T | Witoelar | 4 | 11676144 | T | C | 0·053 | 0·016 | 8·40E-04 | 0·287 |
| rs190982 | rs190982.MEF2C_AS1.A | Lambert | 5 | 88223420 | A | G | 0·056 | 0·016 | 2·81E-04 | 0·611 |
| rs9271192 | rs9271192.HLA_DQA1/HLA_DRB1.C | Lambert | 6 | 32578530 | A | C | -0·092 | 0·017 | 9·02E-08 | 0·73 |
| rs75932628 | rs75932628.TREM2.T | Kunkle | 6 | 41129252 | T | C | 0·699 | 0·1 | 2·95E-12 | 0·008 |
| rs10948363 | rs10948363.CD2AP.G | Lambert | 6 | 47487762 | A | G | -0·083 | 0·016 | 1·77E-07 | 0·724 |
| rs2718058 | rs2718058.GPR141/EPDR1.A | Lambert | 7 | 37841534 | A | G | 0·054 | 0·015 | 2·19E-04 | 0·631 |
| rs1476679 | rs1476679.ZCWPW1.T | Lambert | 7 | 100004446 | T | C | 0·065 | 0·016 | 5·94E-05 | 0·712 |
| rs11771145 | rs11771145.EPHA1_AS1.G | Lambert | 7 | 143110762 | A | G | -0·07 | 0·015 | 4·82E-06 | 0·342 |
| rs28834970 | rs28834970.PTK2B.C | Lambert | 8 | 27195121 | T | C | -0·092 | 0·015 | 1·58E-09 | 0·634 |
| rs9331896 | rs9331896.CLU.T | Lambert | 8 | 27467686 | T | C | 0·127 | 0·016 | 3·62E-16 | 0·613 |
| rs7920721 | rs7920721.RP11_138I18.2/RP11_138I18.1.G | Kunkle | 10 | 11720308 | A | G | -0·078 | 0·015 | 1·94E-07 | 0·61 |
| rs10838725 | rs10838725.CELF1.C | Lambert | 11 | 47557871 | T | C | -0·065 | 0·015 | 1·91E-05 | 0·68 |
| rs983392 | rs983392.AP001257.1/MS4A2.A | Lambert | 11 | 59923508 | A | G | 0·115 | 0·015 | 4·76E-15 | 0·594 |
| rs10792832 | rs10792832.RNU6_560P/RP11_12D16.2.G | Lambert | 11 | 85867875 | A | G | -0·12 | 0·015 | 7·56E-16 | 0·359 |
| rs11218343 | rs11218343.SORL1.T | Lambert | 11 | 121435587 | T | C | 0·205 | 0·037 | 2·63E-08 | 0·96 |
| rs17125944 | rs17125944.FERMT2.C | Lambert | 14 | 53400629 | T | C | -0·12 | 0·025 | 1·08E-06 | 0·909 |
| rs10498633 | rs10498633.SLC24A4.G | Lambert | 14 | 92926952 | T | G | -0·085 | 0·018 | 1·27E-06 | 0·217 |
| rs593742 | rs593742.RP11_30K9.7/ADAM10.A | Kunkle | 15 | 59045774 | A | G | 0·065 | 0·016 | 3·07E-05 | 0·705 |
| rs7185636 | rs7185636.IQCK.T | Kunkle | 16 | 19808163 | T | C | 0·079 | 0·019 | 2·47E-05 | 0·82 |
| rs62039712 | rs62039712.R5SP431/RP11_467I17.1.A | Kunkle | 16 | 79355857 | A | G | 0·153 | 0·029 | 1·17E-07 | 0·116 |
| rs7225151 | rs7225151.RP11_333E1.1/SCIMP.A | Moreno-Grau | 17 | 5137047 | A | G | 0·083 | 0·022 | 1·31E-04 | 0·122 |
| rs138190086 | rs138190086.RP11_269G24.6/CTD_2501B8.5.A | Kunkle | 17 | 61538148 | A | G | 0·254 | 0·057 | 7·46E-06 | 0·02 |
| rs4147929 | rs4147929.ABCA7.A | Lambert | 19 | 1063443 | A | G | 0·122 | 0·02 | 9·56E-10 | 0·182 |
| rs429358 | rs429358.APOE.C | Lambert | 19 | 45411941 | T | C | -1·202 | 0·019 | 0·00E+00 | 0·784 |
| rs3865444 | rs3865444.CD33/SIGLEC22P.C | Moreno-Grau | 19 | 51727962 | A | C | -0·08 | 0·016 | 3·93E-07 | 0·308 |
| rs7274581 | rs7274581.CASS4.T | Lambert | 20 | 55018260 | T | C | 0·115 | 0·026 | 9·02E-06 | 0·911 |
| rs2830500 | rs2830500.ADAMTS1/CYYR1.C | Kunkle | 21 | 28156856 | A | C | -0·081 | 0·016 | 2·53E-07 | 0·308 |

**
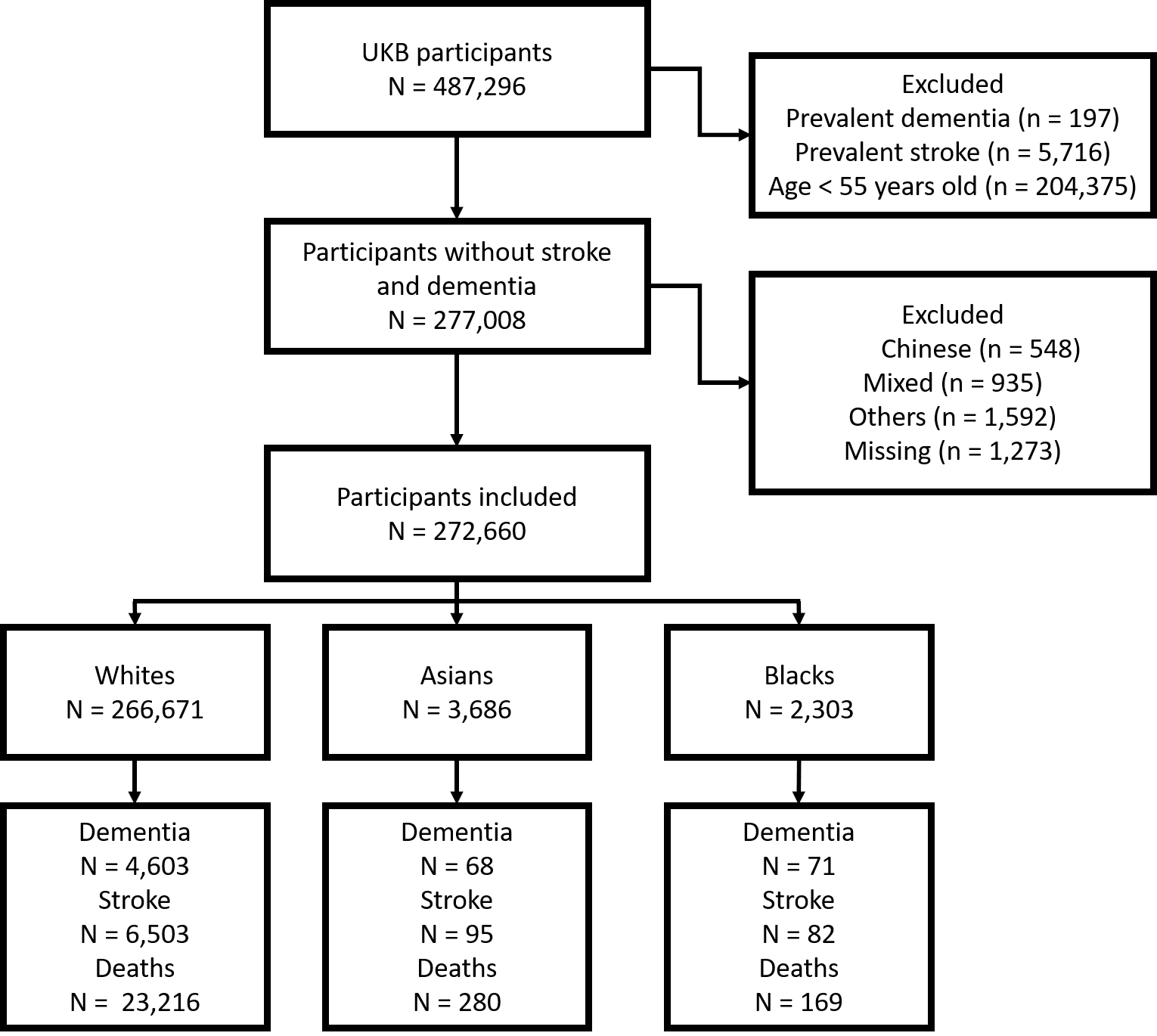
**

**Supplementary Figure 1: Flow of the study participants**

**
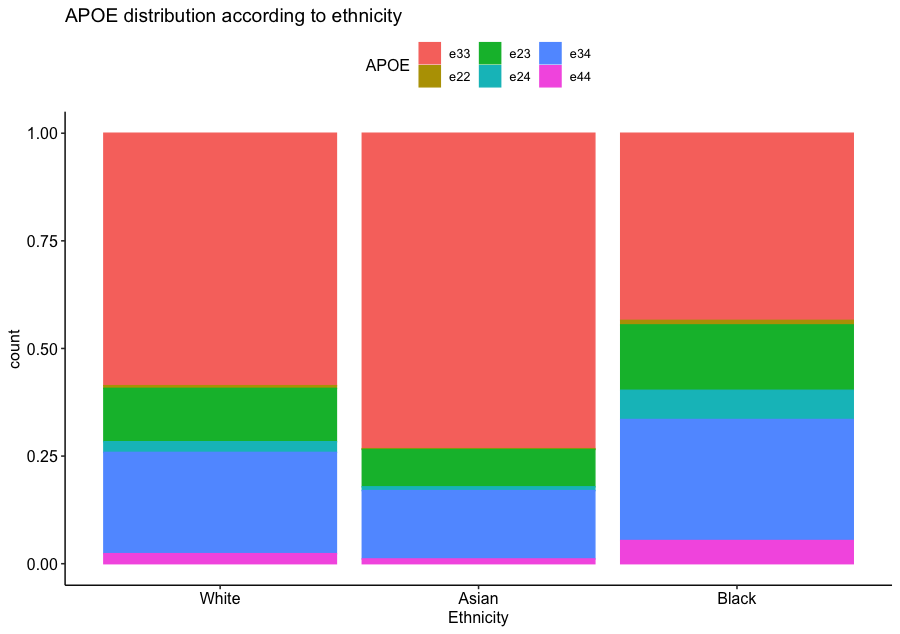
**

**Supplementary Figure 2: Breakdown of APOE genotype by ethnicity.**

**
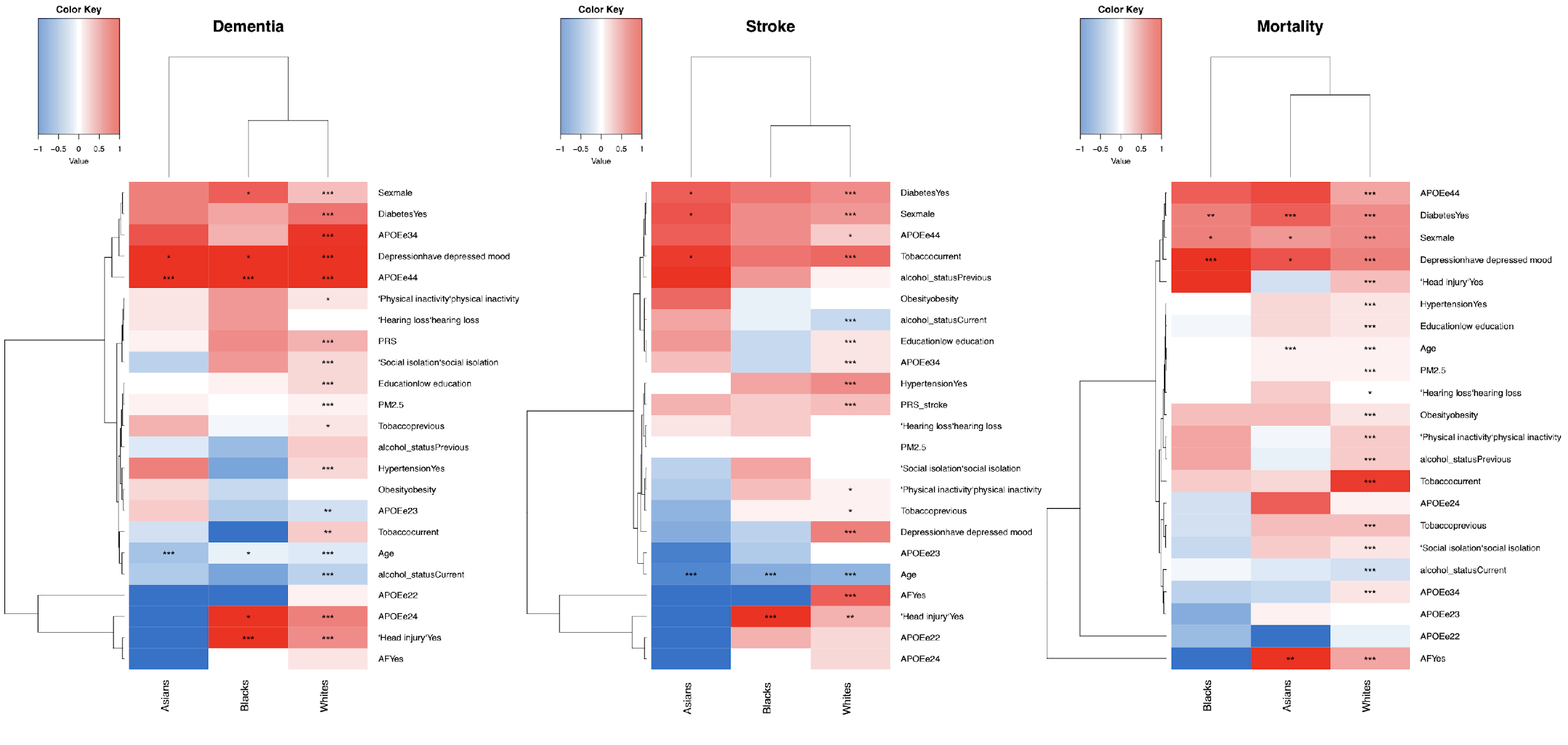
**

**Supplementary Figure 3: Heatmaps of risk factors by ethnicity for dementia, stroke and mortality**
